# Predictive value of ultrasonic artificial intelligence in placental characteristics of early pregnancy for gestational diabetes mellitus

**DOI:** 10.3389/fendo.2024.1344666

**Published:** 2024-03-13

**Authors:** Huien Zhou, Wanming Chen, Chen Chen, Yanying Zeng, Jialin Chen, Jianru Lin, Kun He, Xinmin Guo

**Affiliations:** ^1^ Department of Ultrasound, Guangzhou Red Cross Hospital (Guangzhou Red Cross Hospital of Jinan University), GuangZhou, China; ^2^ Department of Ultrasound, Tianhe District Maternal and Child Hospital of Guangzhou, GuangZhou, China

**Keywords:** gestational diabetes mellitus, placenta, ultrasound imaging, early pregnancy, machine learning, deep learning

## Abstract

**Background:**

To explore the predictive value of placental features in early pregnancy for gestational diabetes mellitus (GDM) using deep and radiomics-based machine learning (ML) applied to ultrasound imaging (USI), and to develop a nomogram in conjunction with clinical features.

**Methods:**

This retrospective multicenter study included 415 pregnant women at 11-13 weeks of gestation from two institutions: the discovery group from center 1 (n=305, control group n=166, GDM group n=139), and the independent validation cohort (n=110, control group n=57, GDM group n=53) from center 2. The 2D USI underwent pre-processed involving normalization and resampling. Subsequently, the study performed screening of radiomics features with Person correlation and mutual information methods. An RBF-SVM model based on radiomics features was constructed using the five-fold cross-validation method. Resnet-50 as the backbone network was employed to learn the region of interest and constructed a deep convolutional neural network (DLCNN) from scratch learning. Clinical variables were screened using one-way logistic regression, with P<0.05 being the threshold for statistical significance, and included in the construction of the clinical model. Nomogram was built based on ML model, DLCNN and clinical models. The performance of nomogram was assessed by calibration curves, area under the receiver operating characteristic curve (AUC) and decision curve analysis (DCA).

**Results:**

The AUCs for the ML model in the discovery cohort and independent validation cohort were 0.91 (0.88-0.94) and 0.86 (0.79-0.93), respectively. And 0.65 (0.59-0.71), 0.69 (0.59-0.79) for the DLCNN, 0.66 (0.59-0.72), 0.66 (0.55-0.76) for the clinical model, respectively. The nomogram exhibited the highest performance with AUCs of 0.93 (0.90-0.95) and 0.88 (0.81-0.94) The receiver operating characteristic curve (ROC) proved the superiority of the nomogram of clinical utility, and calibration curve showed the goodness of fit of the model. The DCA curve indicated that the nomogram outperformed other models in terms of net patient benefit.

**Conclusions:**

The study emphasized the intrinsic relationship between early pregnancy placental USI and the development of GDM. The use of nomogram holds potential for clinical applications in predicting the development of GDM.

## 1 Introduction

Gestational diabetes mellitus (GDM) is a condition characterized by the onset of diabetes during pregnancy The International Diabetes Federation (IDF) reports an annual increase in the global prevalence of GDM (1). GDM development may elevate the risk of metabolic diseases in both mothers and offspring (2, 3). During GDM pregnancies, thickening of the basement membrane of trophoblast cells, increased release of inflammatory cytokines by placental macrophages, and edema of the interchorionic layer of the villi result in reduced placental perfusion (4). Pregnant women at risk for GDM should undergo early screening to detect and manage the condition in a timely manner (5).However, there are no reliable boundaries to predict the development of GDM. In addition to laboratory tests, various imaging technology techniques are available to assess placental function in early pregnancy, including Uterine Artery Pulsatility Index (UtA-PI), free-breathing Magnetic Resonance Imaging (MRI), and three-dimensional placental imaging. However, the assessment of placental function in the context of placental changes in GDM has not been extensively studied (6–8).

The utilization of ultrasound in artificial intelligence (AI) holds significant potential for development. Ultrasound (US) is extensively employed for disease diagnosis owing to its high safety and efficiency. In recent years, with the widespread adoption of Picture Archiving and Communication System (PACS) systems, ultrasound images can now be digitally stored systematically, facilitating the rapid advancement of artificial intelligence (9).Deep learning and machine learning (ML) techniques enable quantitative analysis of images, extracting a wealth of information (10, 11). For instance, Ultrasound images can assess morphological features of tumors, hemodynamic parameters, and elastic properties of tissues, leading to more accurate predictions for tumor diagnosis and treatment (12).

Recently, there has been considerable focus on placental image analysis in the investigation of GDM. The placenta is a vital organ that plays a crucial role in the development and health of the fetus during pregnancy. GDM disrupts placental functioning, adversely affecting the health of both the fetus and the mother. Analyzing placental texture for early prediction of GDM risk carries significant research background and significance (13–16).Placental texture analysis is a non-invasive method that quantifies the structural characteristics of the placenta by extracting textural features from placental images using computer analysis techniques (17).Furthermore, changes in placental ultrasound images can offer detailed information on placental structure, providing insights into the influence of GDM on placental morphology and tissue structure, and enhancing the understanding of GDM’s pathogenesis (16).Moreover, placental image analysis, with the advantages of non-invasiveness, good reproducibility, and no radiation exposure, can serve as a convenient, safe, and feasible means of clinical assessment. It proves to be an effective tool for monitoring the impact of GDM and forming a foundation for the development of individualized treatment and management strategies. Through early prediction, timely interventions can be taken to control blood glucose levels and reduce the incidence of adverse pregnancy outcomes and child health problems.

In conclusion, the aim of this study is to establish a robust prediction model based on early pregnancy indicators for identifying the occurrence of GDM and guiding clinical practice.

## 2 Materials and methods

### 2.1 Inclusion exclusion criteria

**Figure 1:**
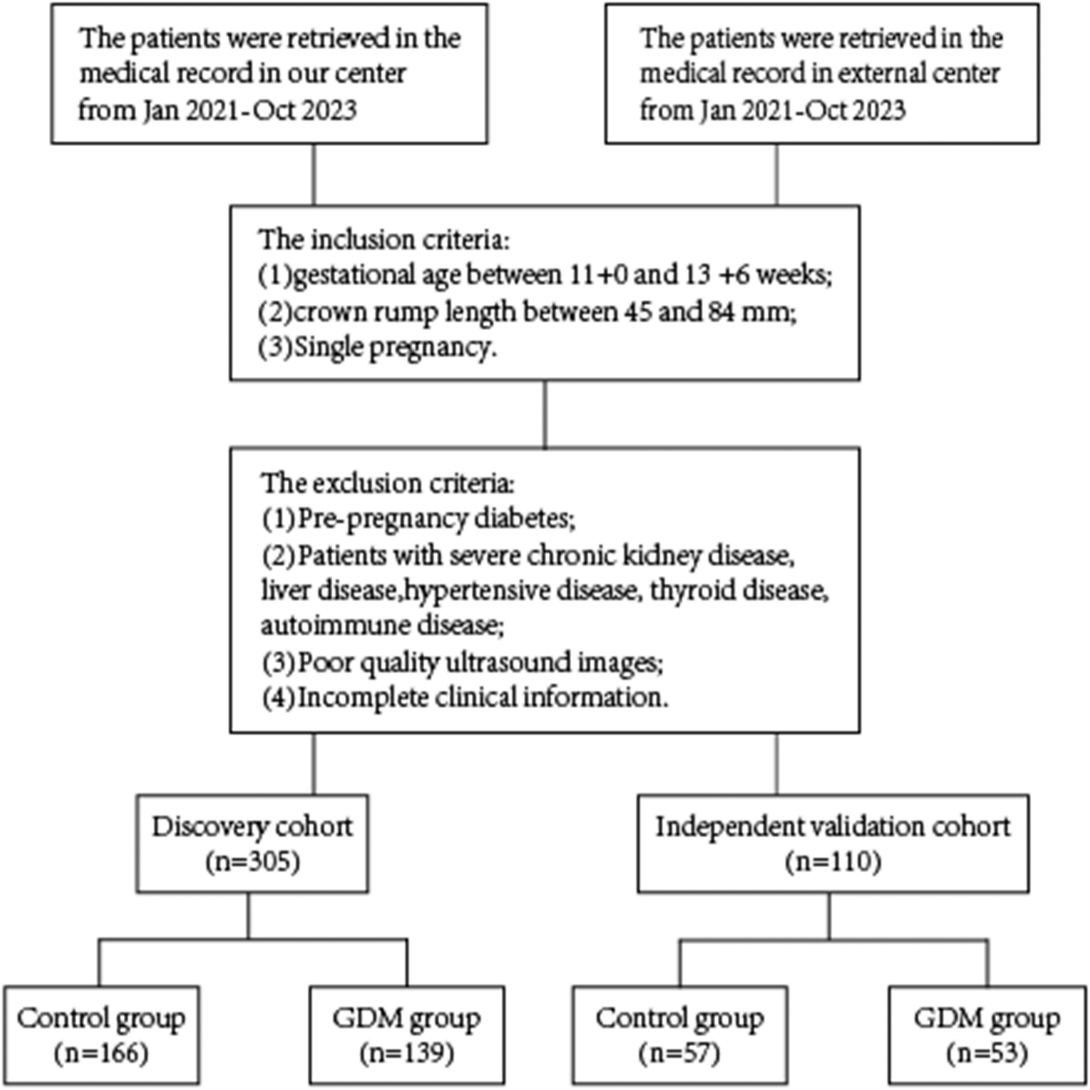
Flowchart for selecting the study population.

Women who underwent early nuchal translucency examinations from January 2021 to October 2023 during pregnancy were retrospectively analyzed. Centre I was located at Guangzhou Red Cross Hospital, and Centre II was based at Tianhe District Maternal and Child Hospital of Guangzhou. In Center 1, 305 pregnant women were enrolled, while Center 2 enrolled 110 pregnant women. Inclusion criteria were: (1) gestational age between 11 + 0 and 13 + 6 weeks; (2) fetal crown-rump length (CRL) between 45 and 84 mm; and (3) singleton pregnancy. Exclusion criteria were: (1) pre-pregnancy diabetes mellitus; (2) a combination of severe cardiovascular, renal, and autoimmune diseases; (3) poor image quality; and (4) incomplete clinical data. The study received approval from the Ethics Committee of Guangzhou Red Cross Hospital and Tianhe District Maternal and Child Hospital of Guangzhou (**Figure 1**) (2024-023-01).

### 2.2 Definition

Pre-pregnancy body mass index (BMI) was obtained from clinical records and calculated by dividing pre-pregnancy weight by the square of the mother’s height. According to the adult weight standards published by the Chinese Ministry of Health, a BMI < 18.5 kg/m² was defined as underweight, 18.5 kg/m² ≤ BMI < 24 kg/m² was defined as normal weight, 24 kg/m² ≤ BMI < 28 kg/m² was defined as overweight, and a BMI ≥ 28 kg/m² was defined as obese (18).

All participants underwent oral glucose tolerance test (OGTT) screening for gestational diabetes mellitus (GDM). Diagnostic criteria were based on the August 2014 revised Chinese criteria. GDM was diagnosed when the results of the OGTT performed between 24 and 28 weeks of gestation met any one or more of the following criteria: 1) fasting blood glucose ≥ 5.1 mmol/L, 2) 1-hour blood glucose ≥ 10.0 mmol/L, and 3) 2-hour blood glucose ≥ 8.5 mmol/L (19).

### 2.3 Regions of interest outlining and radiomics features extraction

In Center 1, Voluson E8, Voluson E10, and HITACHI HV VISION Preirus ultrasound machines, all equipped with 2-5 MHz convex array probes, were utilized. In Center 2, the SAMSUNG WS80A ultrasound machine with a 2-5 MHz convex array probe was employed. Ultrasound scanning adhered to ISUOG practice guidelines.

Ultrasound images of the placenta were obtained during routine early pregnancy antenatal ultrasound examinations. Maximal section images of the placenta were captured, and placental thickness was measured. The images were then saved, and ultrasound images stored in the diagnostic ultrasound system were exported in Joint Photographic Experts Group (JPEG) format for subsequent analysis (20).

### 2.4 Ultrasound images

ROIs were outlined by a physician with 5 years of experience in gynecological ultrasound. ROIs were defined along the edges of the uterus, encompassing the placenta, myometrium, amniotic fluid, and fetal tissues. A senior physician with 10 years of experience in gynecological ultrasound completed the review. In cases of disagreement between the two physicians, resolution was achieved through negotiation. Importantly, the sonographer responsible for outlining and reviewing had no knowledge of the patient’s ultrasound report or pathological details.

Before extracting radiomics features from the ultrasound images, pre-processing steps were undertaken. This involved discretizing image pixels into 30 bandwidth ranges, resampling the image to a 1×1mm² range size, and normalizing image pixels. Various feature types were extracted, encompassing 2D shape features characterizing geometric parameters, intensity features for pixel grey levels, and grey level co-occurrence matrices (GLCM), grey level size zone matrices (GLSZM), grey level run length matrices (GLRLM), neighboring grey tone difference matrices (NGTDM) and grey level dependence matrices (GLDM) characterizing image texture. In addition to the original features, texture features processed by various filtering methods were also extracted. These included Laplacian of Gaussian (LoG), Wavelet, Square, Square root, Logarithm, Exponential, Gradient, and Local Binary Pattern 2D (LBP2D).

### 2.5 Feature screening and ML model construction

Feature screening was conducted on the Discovery cohort. Before screening, the radiomics were normalized to the z-score range (Equation 1). This normalization aimed to eliminate potential effects caused by differences in data magnitudes and to enhance the convergence and computation speed of the model.


(1) $$\text{z}=\frac{\text{x}-\mu }{\sigma }$$

Given the potential impact of high feature correlation on model generalization performance, features were initially assessed using the Pearson correlation test, and those with r > 0.9 were excluded. To evaluate interdependence, the mutual information method, a filtered feature screening approach, was employed. Features with higher mutual information provide more valuable information, reflecting both linear and non-linear relationships.

Considering the dataset’s size, the Radial Basis Function kernel Support Vector Machine (RBF-SVM) is known for its good performance with small-scale data. Therefore, it was selected as the classifier for modeling and validating the radiomics features dataset. Model parameters were tuned and assessed on the discovery cohort using a five-fold cross-validation method. Subsequently, the generalization performance of the well-trained model was evaluated using an independent validation cohort.

### 2.6 DLCNN construction

Before constructing the deep learning model, we algorithmically extracted the lesion region of interest to maximize the model’s ability to learn valid information in the ROIs. Resnet50 served as the backbone network, and we employed a learning-from-scratch strategy by resizing the image to 224×224 pixels and normalizing it. To mitigate overfitting, multiple data augmentation were applied, including random horizontal flip, random vertical flip, random rotation, random grayscale transformation, and transformation of image attributes (brightness, contrast, saturation, and hue).

Upon training, optimal hyperparameters were determined: Batch size of 128, Epochs of 500, Learning rate of 3e-4, Loss function of cross-entropy, Optimizer of Adaptive Moment Estimation (Adam) with β1 of 0.9, β2 of 0.999, epsilon of 1e-8, and weight decay of 0.01. L2 regularization was increased to prevent overfitting.

### 2.7 Clinical modelling and nomogram construction

One-way logistic regression was used to assess valuable clinical variables on the discovery cohort, P< 0.05 was considered statistically different and thus included in the logistic regression model for the construction of the clinical model.

Nomogram was constructed based on the combination of ML model of radiomics, DLCNN and clinical model. The model’s goodness of fit P >0.05 was considered that nomogram had a good degree of fit in terms of actual observations and predicted probabilities directly.

**Figure 2:**
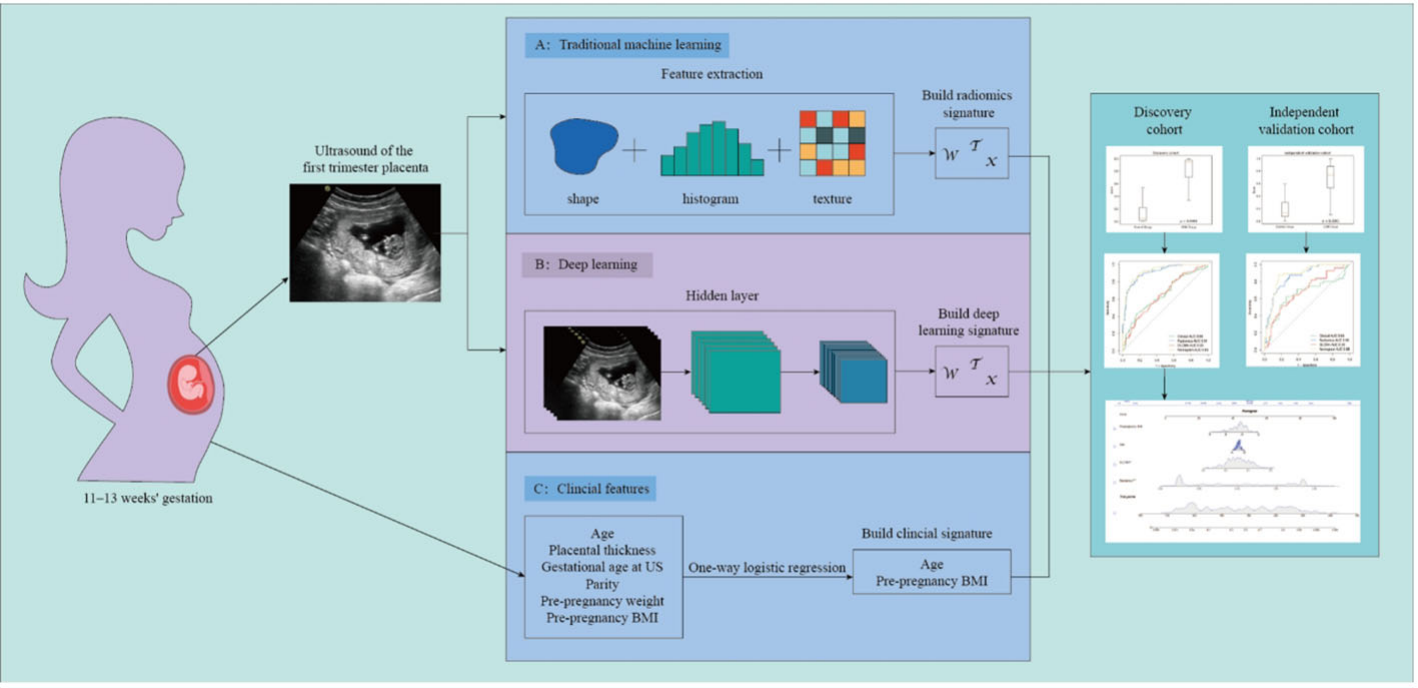
Workflow of this study.

**Figure 2** illustrates the process of studying image segmentation and analysis used in this study.

### 2.8 Statistical analysis

Statistical analyses were conducted using R software (version 4.2.2, https://www.r-project.org/). For continuous variables, the Shapiro-Wilk test was employed for distribution tests. If the data adhered to a normal distribution, parametric tests were utilized; otherwise, non-parametric tests were applied. Categorical variables were analyzed using the chi-square test or Fisher’s exact test (if applicable). Results for categorical and continuous variables were presented as frequencies (percentages), mean ± standard deviation, or median (interquartile range), as appropriate. Effect size and sample size were calculated by statistical methods. f=0.4, a=0.05, 1-β=0.8, and the required sample size was 52 people.

The predictive performance of the model was assessed using AUC and its 95% confidence interval. Target area outlining, radiomics features extraction, feature screening, and ML model construction were carried out using the open-source toolkit OpenCV (version 4.8.1, https://opencv.org/), PyRadiomics (version 3.1.0, https://pyradiomics.readthedocs.io/), and Scikit-learn (version 1.0.2, https://scikit-learn.org/). Deep convolutional network modeling was implemented based on the PyTorch framework (version 1.13.0, https://pytorch.org/).

## 3 Results

### 3.1 Clinical baseline and clinical model construction

A total of 415 patients were enrolled, with 305 in the Discovery cohort and 110 in the Independent Validation cohort. Baseline demographic characteristics, including Age, Placental thickness in early pregnancy, NT gestational week, Parity, Pre-pregnancy weight, Pre-pregnancy BMI, and others, are presented in **Table 1**. No significant differences were observed between the two groups in early pregnancy placental thickness, NT test gestational week, parity, and pre-pregnancy weight (P > 0.05).

**Table 1:** Patient characteristics^a^.

	Discovery cohort (N=305)			Independent validation cohort (N=110)		
	Control group (N=166)	GDM group (N=139)	P-value	Control group (N=57)	GDM group (N=53)	P-value
Age (years)	31.1 ± 4.36	32.1 ± 4.02	0.04	29.6 ± 3.78	31.3 ± 4.12	0.03
Parity			0.31			0.27
Primiparous	80 (48.2%)	76 (54.7%)		35 (61.4%)	26 (49.1%)	
Multiparous	86 (51.8%)	63 (45.3%)		22 (38.6%)	27 (50.9%)	
Pre-pregnancy weight (kg)	54.6 ± 9.49	56.4 ± 10.00	0.11	51.5 ± 7.17	55.5 ± 9.88	0.02
Pre-pregnancy BMI (kg/m²)	21.4 ± 3.48	22.3 ± 3.69	0.03	20.2 ± 2.08	21.9 ± 3.84	0.00
Placental thickness (mm)	13.8 ± 2.42	14.3 ± 3.03	0.12	13.5 ± 1.71	13.9 ± 1.94	0.26
Gestational age at US(weeks)	11.7 ± 0.65	11.8 ± 0.62	0.29	11.9 ± 0.68	12.3 ± 1.38	0.06

a Data were presented as No. (%) and mean ± SD.

Results from one-way logistic regression analysis indicated significant differences in age and pre-pregnancy BMI between the groups (P< 0.05). The GDM group exhibited higher age and pre-pregnancy BMI, leading to their inclusion in the multifactorial logistic regression model for constructing the clinical model. The AUC, sensitivity, specificity, and accuracy of the clinical model for the Discovery cohort were 0.66 (95% CI 0.59-0.72), 0.44, 0.83, 0.65, and 0.54, respectively. In the Independent Validation cohort, these metrics were 0.66 (95% CI 0.55-0.76), 0.27, 0.93, 0.59, and 0.40, respectively.

### 3.2 Feature screening and model building and validation

**Figure 3:**
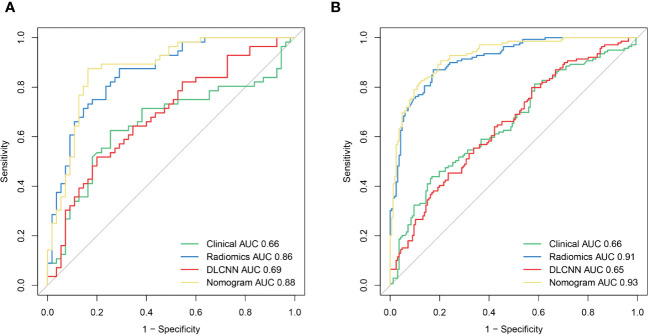
The ROCof the Clinical model, Radiomics model, DLCNN model, and Nomogram model in the training **(A)** and validation **(B)** cohorts, respectively.

**Figure 4:**
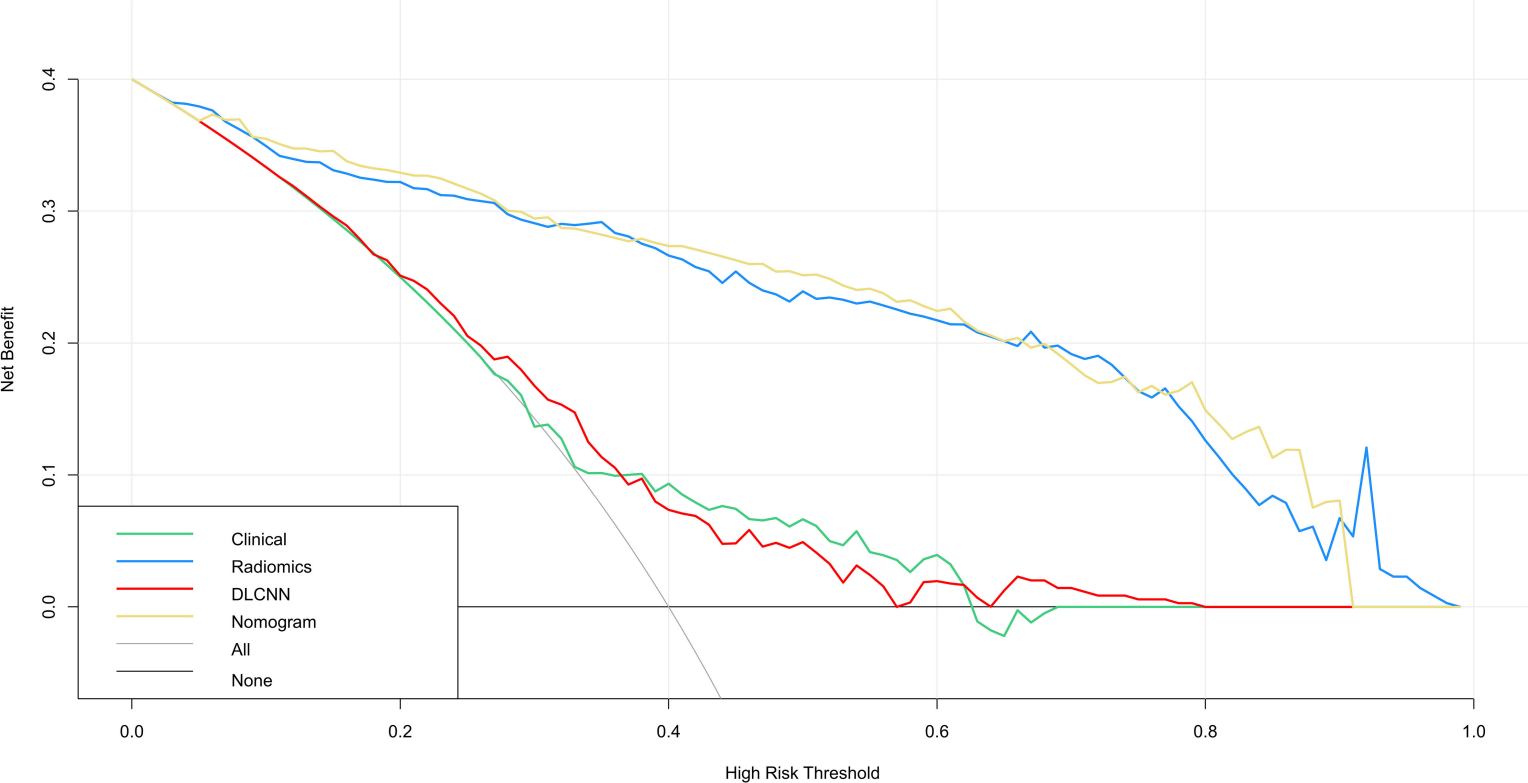
DCA for four models predicting GDM. The graphs show that the nomogram model has the greatest net benefit.

**Figure 3** illustrates the ROC curves for the Discovery cohort and Independent Validation cohort. The DCAs of the four models are shown in **Figure 4**, and **Table 2** presents the diagnostic performance of each model.

**Table 2:** Diagnostic performance of different models for predicting GDM in training and test cohorts.

Model	Discovery cohort					Independent validation cohort				
	AUC (95%CI)	Sen	Spe	Acc	F1	AUC (95%CI)	Sen	Spe	Acc	F1
Clinical	0.66 (0.59-0.72)	0.44	0.83	0.65	0.54	0.66 (0.55-0.76)	0.27	0.93	0.59	0.40
DLCNN	0.65 (0.59-0.71)	0.80	0.43	0.60	0.64	0.69 (0.59-0.79)	0.96	0.07	0.52	0.67
Radiomics	0.91 (0.88-0.94)	0.85	0.83	0.91	0.83	0.86 (0.79-0.93)	0.75	0.82	0.78	0.78
Nomogram	0.93 (0.90-0.95)	0.91	0.80	0.85	0.84	0.88 (0.81-0.94)	0.88	0.78	0.83	0.84

AUC, area under the receiver operating characteristic curve; Sen, sensitivity; Spe, specificity; F1, F1-score.

A total of 1311 ultrasound radiomics features were extracted, categorized into 252 FirstOrderStatistics, 336 GLCM, 196 GLDM, 224 GLRLM, 224 GLSZM, 70 NGTDM, and 9 Shape-based (shape2D).

Using the person correlation coefficient and mutual information as filtered feature selection methods, 38 features with predictive value were selected to build the ML model. These included one Shape-based feature, 12 FirstOrderStatistics, 15 Glcm, 3 Gldm, 2 Glrlm, and 6 Ngtdm. Employing the RBF-SVM to construct the model, the AUC, sensitivity, specificity, and accuracy for the Discovery cohort were 0.91 (95% CI 0.88-0.94), 0.85, 0.83, 0.91, and 0.83, respectively. In the Independent Validation cohort, these metrics were 0.86 (95% CI 0.79-0.93), 0.75, 0.82, 0.78, and 0.78, respectively.

For the Resnet50 model, the AUC, sensitivity, specificity, and accuracy in the Discovery cohort were 0.65 (95% CI 0.59-0.71), 0.80, 0.43, 0.60, and 0.64, respectively. In the Independent Validation cohort, these metrics were 0.69 (95% CI 0.59-0.79), 0.96, 0.07, 0.52, and 0.67, respectively.

**Figure 5:**
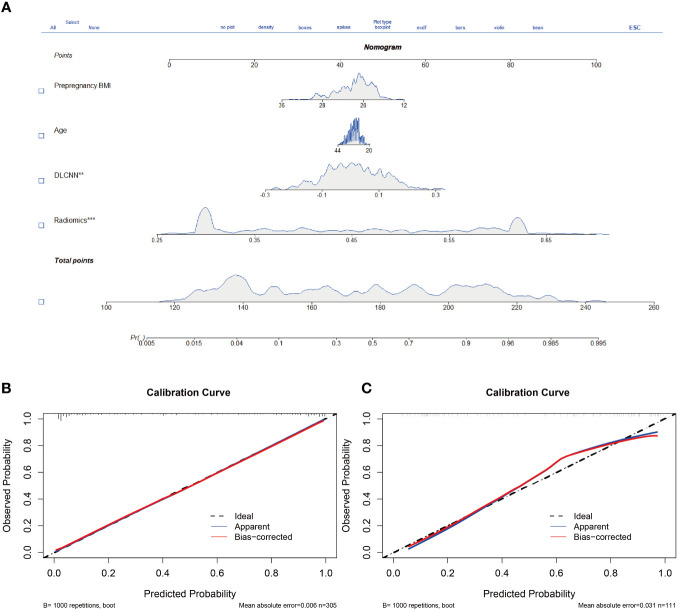
Nomogram and calibration curves. **(A)** The nomogram, combining Pre-pregnancy BMI、age、DLCNN score and Rad-score, was developed in Discovery cohort. **(B, C)** The nomogram calibration curves in Discovery cohort **(B)** and Independent validation cohort **(C)**. Calibration curves indicate the goodness-of-fit of the model. ** means p<0.01. *** means p<0.001.

Additionally, we evaluated a nomogram combining ML, DLCNN, and clinical model (**Figure 5A**). **Figure 5B** and **Figure 5C** shows the consistency of predicted and observed probabilities between the Discovery cohort and Independent Validation cohort nomogram models. In the Discovery cohort, the nomogram exhibited the highest discrimination with AUCs of 0.951 (95% CI 0.87-1.00) and 0.893 (95% CI 0.75-0.97) for the Discovery and Independent Validation cohorts, respectively. Sensitivities were 0.91 and 0.80, specificity 0.88 and 0.78, and accuracy 0.85 and 0.83, respectively. The DCAs show that the nomogram model has the greatest net benefit.

## 4 Discussion

The field of Artificial Intelligence is developing rapidly, and its advantages in the field of medical image recognition and processing continue to emerge and are gradually being used in all aspects of obstetrics and gynaecology (21, 22). Our study establishes a nomogram that incorporates two clinical factors, radiomics features, and DLCNN. This nomogram yielded a higher AUC than a single model, demonstrating its excellent clinical application. It can further aid in treatment decisions and improve clinical outcomes.

Advanced age during pregnancy is a widely recognized major risk factor for GDM. This study revealed that maternal age was higher in the GDM group than in the non-GDM group. Maternal age was subsequently included in the ROC analysis (23). A systematic evaluation and meta-analysis of over 120 million participants indicated a linear relationship between GDM risk and maternal age. Another study demonstrated that the risk of GDM increases with age, with a 12.5 percent rise in GDM risk for each additional year of age (24). This aligns with the findings of our study. We hypothesize that as pregnant women age, their body’s metabolic capacity decreases, leading to insufficient insulin secretion and pancreatic β-cell hypofunction (25).

Additionally, obesity is a recognized risk factor for GDM. The results of this study showed that the pre-pregnancy BMI of pregnant women with GDM was higher than that of pregnant women with normal pregnancies. Maria Mirabelli et al. found that maternal pre-pregnancy obesity was associated with almost three times the risk of GDM (26). M. R. Torloni demonstrated that the prevalence of GDM increased with BMI, and the risk of GDM was positively correlated with pre-pregnancy BMI (27). J. Ogonowski concluded that the risk of GDM increases not only in overweight but also in normal-weight women, making pre-pregnancy BMI a predictor of gestational diabetes (28). Therefore, controlling pre-pregnancy BMI and managing weight gain during pregnancy are crucial, and effective preventive interventions for weight must commence as early as possible.

Advanced age and pre-pregnancy obesity are identified as risk factors, but their accuracy in predicting GDM is unsatisfactory. In this study, we investigated the relationship between placental ultrasound images in early pregnancy and the development of GDM. We found that nomogram for predicting the development of GDM, are potentially useful for clinical applications. This finding may be related to a series of changes in placental texture characteristics in pregnant women with GDM that occur early in pregnancy. Many studies have shown that placental morphology undergoes changes in early pregnancy, including alterations in surface area and volume, as well as histological changes such as an increased volume of the interstitium and terminal villi of the villous structure, placental vascular function, and vascular lesions, the number of syncytiotrophoblast junctions, fibrillar areas, and glycogen deposition (5, 29).

Obinna et al. demonstrated that the GLCM, GLRLM, and histogram parameters were able to differentiate between normal and abnormal placental tissue by analyzing the textural features of 500 ultrasound placental images (p-value < 0.05) (15).Hongshuang Sun MD et al. developed a model to distinguish placental features in ultrasound images of GDM patients and healthy pregnant women, confirming significant changes in abnormal placental features during diabetic pregnancy (10). The results consistently showed that ML and deep learning can effectively distinguish the textural features of the placenta in early pregnancy in patients with GDM, which is an important diagnostic marker for predicting GDM.

Currently, OGTT stands as the gold standard for diagnosing GDM. Consequently, GDM is typically identified during the middle and late stages of pregnancy. Unfortunately, by this point, the risk of hyperglycemia to both the mother and fetus becomes irreversible. Gestational Diabetes Australia recommends screening for GDM in high-risk women before 24 weeks of gestation, aiming for early diagnosis and control of normoglycemia in pregnant women to minimize adverse outcomes (30). However, this recommendation lacks robust supporting evidence. Therefore, it becomes imperative to develop reliable prediction models in early pregnancy to identify high-risk pregnant women for GDM.

Between 11 and 14 weeks of gestation in GDM, three-dimensional ultrasound failed to reveal significant differences in placental volume (31). However, M. Şengül’s study, investigating real-time placental strain elastography in early pregnancy, identified the placental strain ratio as an independent risk factor for GDM development (32). It showed that gestational diabetic patients exhibited higher placental stiffness, suggesting a series of changes in the pathological histology and morphological structure of the placenta during early pregnancy (33). These alterations may lead to functional changes in the placenta, impacting the health of both the pregnant woman and the fetus (34).

Predicting GDM risk in pregnant women by identifying early pregnancy placental features through radiomics and deep learning methods could enable interventions and lifestyle changes to prevent GDM development (35). This approach also provides clinicians with sufficient time to formulate appropriate therapeutic strategies, reducing the harm associated with GDM or preventing its occurrence. However, our study has limitations. The fundamental requirement of artificial intelligence is learning from a large dataset. The small sample size in this study, may explain the low specificity, as a limited dataset fails to capture the diversity and heterogeneity present in the placenta. Additionally, exploring multimodal data to analyze placental heterogeneity and assess function more precisely is crucial. In future studies, we will further implement multi-center, large-sample clinical trials.

In conclusion, our study not only developed an effective model for differentiating placental features in ultrasound images of GDM patients and healthy pregnant women but also confirmed significant changes in abnormal placental features during early diabetic pregnancy. Obtaining information about placental function holds vital ancillary value for diagnosing and treating gestational diabetes. This advanced modeling approach opens opportunities, suggesting that future research should focus on providing models that offer both the physiological features of a normal pregnancy and aid in clinical decision-making quickly and efficiently.

## Data availability statement

The raw data supporting the conclusions of this article will be made available by the authors, without undue reservation.

## Ethics statement

The studies involving humans were approved by Guangzhou Red Cross Hospital Ethics Committee. The studies were conducted in accordance with the local legislation and institutional requirements. Written informed consent for participation was not required from the participants or the participants’ legal guardians/next of kin in accordance with the national legislation and institutional requirements.

## Author contributions

HZ: Writing – original draft. WC: Writing – review & editing. CC: Writing – review & editing. YZ: Writing – review & editing. JC: Writing – review & editing. JL: Writing – review & editing. KH: Writing – review & editing. XG: Writing – original draft.
